# Identification of a Functional Nuclear Localization Signal Mediating Nuclear Import of the Zinc Finger Transcription Factor ZNF24

**DOI:** 10.1371/journal.pone.0079910

**Published:** 2013-11-01

**Authors:** Jian-Zhong Li, Xia Chen, Xue-Lian Gong, Hong-Yuan Hu, Duo Shi, Yi-Ming Lu, Lei Qiu, Fa Lu, Zhen-Lin Hu, Jun-Ping Zhang

**Affiliations:** 1 Department of Biochemical Pharmacy, Second Military Medical University, Shanghai, China; 2 Cancer Institute, Second Military Medical University, Shanghai, China; 3 Department of Health Toxicology, Second Military Medical University, Shanghai, China; Université Paris-Diderot, France

## Abstract

ZNF24 is a member of the SCAN domain family of Krüppel-like zinc finger (ZF) transcription factors, which plays a critical role in cell proliferation and differentiation. However, how ZNF24 enters the nucleus in order to exert its function remains unclear since its nuclear localization signal(s) (NLS) has not been identified. Here, we generated a series of GFP-tagged deletion and point mutants and assessed their subcellular localization. Our results delimit the NLS to ZF1-2. Deletion of ZF1-2 caused cytoplasmic accumulation of ZNF24. Fusion of the ZF1-2 to green fluorescent protein (GFP) targeted GFP to the nucleus, demonstrating that the ZF1-2 is both necessary and sufficient for nuclear localization. ZNF24 containing histidine to leucine mutations that disrupt the structure of ZF1 or/and ZF2 retains appropriate nuclear localization, indicating that neither the tertiary structure of the zinc fingers nor specific DNA binding are necessary for nuclear localization. K286A and R290A mutation led to partial cytoplasmic accumulation. Co-immunoprecipitation demonstrated that ZNF24 interacted with importin-β and this interaction required the ZF motifs. The β-Catenin (CTNNB1) luciferase assays showed that the ZNF24 mutants defective in nuclear localization could not promote CTNNB1promoter activation as the wild-type ZNF24 did. Taken together, these results suggest that consecutive ZF1-2 is critical for the regulation of ZNF24 nuclear localization and its transactivation function.

## Introduction

 The zinc finger protein 24 (ZNF24, also known as ZNF/Zfp191[1,2] )belongs to the SCAN domain subfamily of Krüppel-like zinc finger （ZF） transcription factors[3]. This gene was initially named as RSG-A (for retinoic acid suppressed gene-A) because its mRNA can be amplified by homologous RT-PCR only in retinoic acid-untreated but not in retinoic acid-treated acute promyelocytic leukemia NB4 cells[2]. ZNF24 contains four continuous typical C_2_H_2_ zinc fingers in its C-terminus, and one SCAN domain in its N-terminus [2,4].Whereas Krüppel-like zinc fingers bind to DNA-specific sequences and are widely represented in all species[5], the SCAN domain participates in protein-protein interactions and has to date only been found in vertebrates [6,7] with a remarked absence in birds. ZNF24 can specifically interact with the widespread TCAT motif which constitutes the HUMTH01 microsatellite in the tyrosine hydroxylase (TH) gene (encoding the rate-limiting enzyme in the synthesis of catecholamines)[8]. Allelic variations of HUMTH01 are known to have a quantitative silencing effect on TH gene expression and to correlate with quantitative and qualitative changes in the binding by ZNF24[8]. ZNF24 also can directly bind to CTNNB1 promoter, and activates the expression of β-Catenin and promotes cell proliferation of hepatocellular carcinoma[9]. ZNF24 shows 94% identity to its mouse homologue Zfp191, which is the most highly conserved among the human-mouse SCAN family member orthologues pairs[10]. The SCAN domain of ZNF24 displays a suppressive effect on the transcription in CHO and NIH3T3 cells[2]. ZNF24 is involved in negative regulation of VEGF and PDGFRB and may represent a novel repressor of VEGF and PDGFRB transcription[11,12]. We have recently shown that ZNF24 is a pleiotropic factor that has a role in hematopoiesis, brain development and cancers[13], but the molecular mechanisms underlying its functions are not clearly understood. Our previous study, gene targeting provided the first evidence that ZNF24 has important functions, because the null mutation caused early embryonic lethality in mice[14]. Recent studies suggest ZNF24 is necessary to maintain neural cells in a cycling progenitor status by preventing them from leaving the cell cycle and being committed into a differentiation pathway[15]. In addition, ZNF24 is required for the myelinating function of differentiated oligodendrocytes[16]. Like many transcription factors, ZNF24 must first enter the nucleus in order to exert its function. How the nuclear localization of ZNF24 is regulated, however, has not been investigated.

Nuclear import is essential for nuclear proteins, such as transcription factors, to execute their function in the nucleus. Nuclear import is regulated by mechanisms largely divided into two categories: free diffusion and active transport. Small proteins (less than 40 kDa) can usually pass through the nuclear pore by the free diffusion mechanism, whereas larger proteins use active transport importing mechanisms [17]. The latter mechanism requires the cargo protein to have nuclear localization signal (NLS). Through the NLS, the cargo protein binds to the nuclear import receptor proteins called importins (importin-β1 in most cases and importin-α/β1 complex in other cases). NLSs are generally grouped into classical and non-classical types. The classical type NLSs include monopartite NLS (mNLS) (a single stretch of basic amino acid (aa) with a consensus sequence of (K/R)_4-6_) and bipartite NLS (two small stretches of basic aa linked by 10-12 aa in between with a consensus sequence of (K/R)_2_X_10-12_(K/R)_3_) [18]. Most non-classical NLSs consist of a single long stretch of non-basic aa (20-40 aa) as in hnRNP[19], HuR [20 ] and IκBα[21]. Some non-classical NLSs are a single short stretch containing one or more basic aa, but distinct from the mNLS, as in Cdc6 [22], FGF2 [23] and Sam68 [24]. Many studies have demonstrated that nuclear localization of proteins is strictly regulated through their post-translational modifications. These modifications include phosphorylation, ubiquitylation, sumoylation, acetylation, methylation and glycosylation[25-28]. Since the subcellular localization may provide valuable information on the function of ZNF24, we set out to further study its cellular localization and to determine its localization domains. 

In this report, we have identified ZNF24 as a nuclear protein and further identified and characterized the NLS of ZNF24. The NLS localizes to ZF1-2 and is both necessary and sufficient for nuclear localization. We also demonstrate that the basic residues 286 and 290 within this region are the critical determinants for nuclear localization. The C-terminal ZF regions of ZNF24 are essential for transactivity of the β-Catenin.

## Materials and Methods

### Plasmid Construction

**Figure 1:**
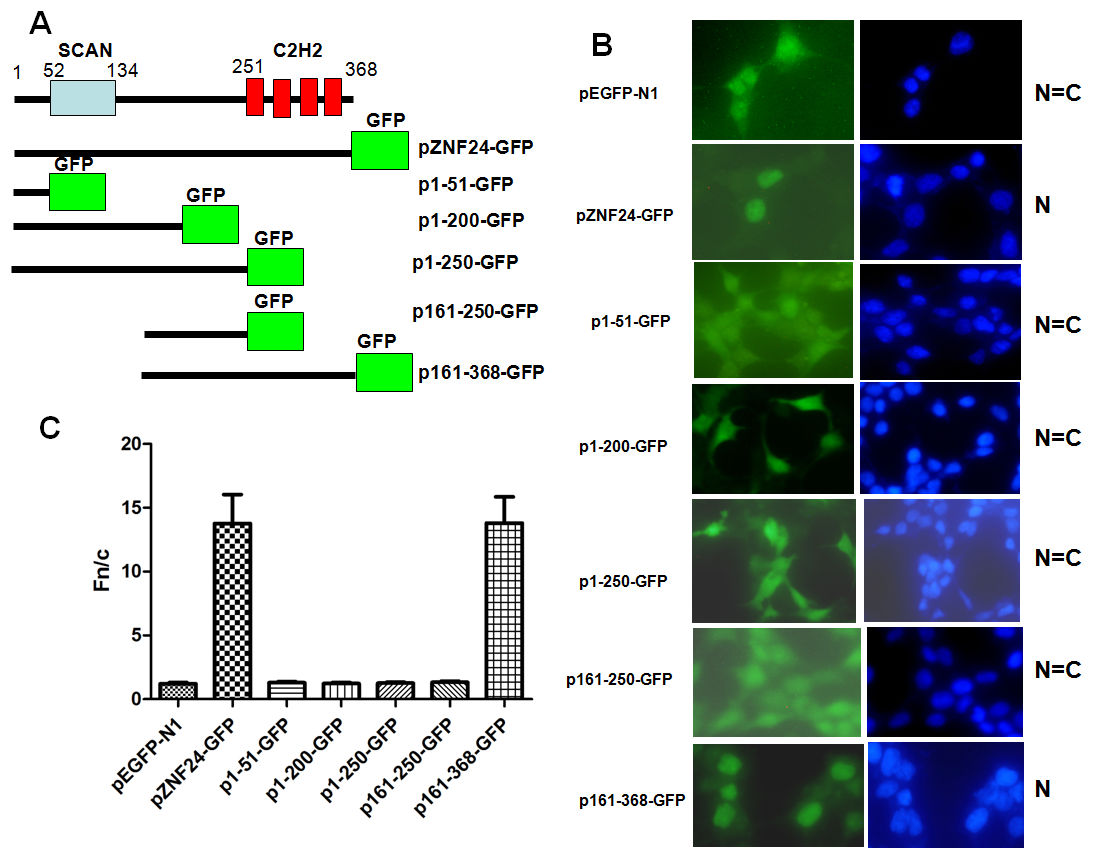
ZNF24 DNA binding domain is necessary for nuclear localization in HEK293 cells. (A) Schematic of full-length and deletion ZNF24-GFP constructs used in this study (not to scale). The DNA binding domain is shown as four red boxes at the carboxyl terminus, with each box depicting a Krüppel-like zinc finger. All deletion constructs were fused to the N terminus of the GFP. (B) Protein localization of the ZNF24 constructs. HEK293 cells transfected with GFP control, pZNF24-GFP, p1-51-GFP, p1-200-GFP, p1-250-GFP, p161-250-GFP or p161-368-GFP. Digital images were taken by fluorescent microscopy. DAPI stain is shown to delineate nuclear boundaries (blue). Patterns of localization are summarized on the right by observing 100 transfected cells from 10 to 15 independent fields. N, exclusively nuclear; C, exclusively cytoplasmic; N=C, most cells show both nuclear and cytoplasmic localization with similar staining intensity in the nucleus and cytoplasm; N<C, most cells show both nuclear and cytoplasmic localization with brighter staining intensity in the cytoplasm; N>C, most cells show both nuclear and cytoplasmic localization with brighter staining intensity in the nucleus. (C) Results (mean±S.E.M., n=100) for quantitative analysis of images to determine the nuclear to cytoplasmic fluorescence ratio (Fn/c).

DNA encoding amino acids 1 to 51 (1 to 200, 1 to 250 and 161-250 ) and 161 to 368 of human ZNF24 were amplified by PCR using appropriate primers together with an upstream Kozak sequence, and the products were cloned into the Xho I/Sac II sites of pEGFP-N1 (Clontech) vector to produce p1-51-GFP (p1-200-GFP, p1-250-GFP and p161-250-GFP ) and p161-368-GFP, respectively(Figure 1A). Full-length human ZNF24 was amplified by PCR and was cloned into the Xho I/Sac II sites of pEGFP-N1 yielding pEGFP-ZNF24. The plasmid p1.5kb-Luc used for promoter reporter assays was made by polymerase chain reaction (PCR) from human genomic DNA, and subcloned into the Kpn I/Hind III sites of luciferase expression vector pGL3 basic (Promega). The primers are as follows: forward, 5’- ggaggtaccGACGGCAGTTGGCATTAC -3’; reverse, 5’-ggaaagcttGCTCCTCAGACCTTCCTCC-3’. The point mutations and internal deletions were carried out using site-directed mutagenesis by overlapping PCR. The primer pairs (forward/reverse) used to generate the point and internal deletion mutants are as follows. K258A: TGAATGTGGAGCACACTTCAGT/ ACTGAAGTGTGCTCCACATCA; S261A: AACACTTCGCTCAGGGCTCA/TG AGCCCTGAGCGAAGTGTT; S264A: AGAATTCACGCTGGGGAGAA/TTCTCC CCAGCGTGAATTCT; R271A: TCTTCATCAAGCAATTCACAGTG/CACTGTG AATTGCTTGATGAAGA; S274A: AGAATTCACGCTGGGGAGAAAC/GTT TCTCCCCAGCGTGAATTCT; K277A: ATTCACAGTGGGGAGGCACCTTATG GATGTGTT/AACACATCCATAAGGTGCCTCCCCACTGTGAAT; Y279A: GTG GGGAGAAACCTGCTGGATGTGTTGAGT/ACTCAACACATCCAGCAGGTTT CTCCCCAC;K286A: TGTGTTGAGTGTGGGGCAGCATTCAGCCGAAG/CTTC GGCTGAATGCTGCCCCACACTCAACACA; S289A: GAAAGCATTCGCCCGA AGTTCC/GGAACTTCGGGCGAATGCTTTC; R290A: GGAAAGCATTCAGCG CAAGTTCCATTCTTGTGC/GCACAAGAATGGAACTTGCGCTGAATGCTTTC C; S291A: TCAGCCGAGCTTCCATTCTT/AAGAATGGAAGCTCGGCTGA; S292A: AGCCGAAGTGCCATTCTTGTG/CACAAGAATGGCACTTCGGCT; R299A: CTTGTGCAACACCAGGCAGTCCACCCGCGGG/CCCGCGGGTGG ACTGCCTGGTGTTGCACA AG; H269/273L: CTTATTCTTCTTCAAAGAATT CTCAGTGGGGA/TCCCCACTGAGAATTCTTTGAAGAAGAATAAG; H297/ 301L: TGTGCAACTCCAGAGAGTCCTCACTGGAGA/TCTCCAGTGAGG ACT CTCTGGAGTTGCACA; dZF1-2: CTCTCGAAAGAAACAAACTGGAGA AAAACCTTA/TAAGGTTTTTCTCCAGTTTGTTTCTTTCGAGAG; dZF1: CTCTCGAAAGAAACAAAGTGGGGAGAAACCTT/AAGGTTTCTCCCCACTT TGTTTCTTTCGAGAG; dZF2: TCATCAAAGAATTCACACTGGAGAAAAACC TT/AAGGTTTTTCTCCAGTGTGAATTCTTTGATGA. The sequences of all constructs were confirmed by DNA sequencing.

### Cell culture and transfection

HEK293 and HeLa cells (obtained from American Type Culture Collection) were maintained in Dulbecco’s modiﬁed Eagle’s medium with 10% fetal bovine serum. Lipofectamine 2000 (Invitrogen) was used for transfection according to the manufacturer’s protocols. 

### Cellular localization studies

 The GFP-plasmid DNA were transiently transfected into HEK293 cells with Lipofectamine 2000 according to the manufacturer’s directions (Invitrogen). At 48 h post-transfection the cells mounted on coverslips were fixed with 4% (w/v) formaldehyde in PBS for 10 min at room temperature, then permeabilized with 0.5% Triton X-100 in PBS for 10 min, and the nuclei stained with 0.01µg/ml DAPI (4′,6′-diamidino-2-phenylindol) for 15 min. Fluorescence was detected with a Nikon Diaphot 200 inverted fluorescence microscope. Digitized images were analysed using the ImageJ2x public domain software (NIH) to determine the ratio of nuclear (Fn) to cytoplasmic (Fc) fluorescence (Fn/c) according to the formula: Fn/c = (Fn Fb)/(Fc−Fb), where Fb is background autofluorescence[25]. Statistical analysis was performed using Welch’s test and the GraphPad Prism 5.0 software.

### Western blotting and co-immunoprecipitation (Co-IP)

 Western blotting was performed essentially as described previously [29]. HEK293 cells were transfected with the ZNF24 constructs for 48 h, washed with ice-cold PBS and lysed with 1× SDS sample buffer. The lysates were used for blotting. The primary antibody used was mouse anti-GFP monoclonal antibody (Santa Cruz) and the secondary antibody was IRDye 800CW-goat-anti-mouse antibody (LI-COR Biosciences). For Co-IP, 500 µg of whole cell lysate was pre-cleared with 5 μl of Protein A/G agarose beads (Santa Cruz) for 30 min at 4°C with rotation. Importin-β1 was IPed from the lysate using 1.0μg of antibody (Karyopherin-β1, Calbiochem) or mouse IgG (Santa Cruz) for 2 h and 20 μl of Protein A/G beads overnight at 4°C. The beads were then washed, boiled in sample buffer and subject to SDS-PAGE gel electrophoresis and western blotting with either the GFP (Santa Cruz) or importin antibody.

### Promoter reporter assays

Luciferase reporter assays were performed essentially as described previously [12]. Briefly, HeLa cells were co-transfected with ZNF24 or its mutants along β-Catenin (CTNNB1) promoter reporter construct (p1.5kb-Luc). Control reporter expressing *Renilla luciferase* was used to normalize transfection efficiencies. Luciferase activity was determined using the dual luciferase reporter assay system (Promega) and the plate-reading luminometer (BioTek Synegy 4).

## Results

### The ZFs are necessary for ZNF24 nuclear localization

The presence of four zinc finger motifs suggests that ZNF24 is likely to be a transcription factor, which would be reflected in a nuclear localization. To determine the subcellular localization of ZNF24, a plasmid containing the ZNF24 cDNA with a GFP tag under the control of the CMV transcriptional regulatory region was constructed. Using fluorescence microscopy we determined the cellular localization pattern of exogenous ZNF24 protein in the human embryo kidney cell line (HEK293), and quantification of the nuclear/cytoplasmic fluorescence intensity ratio was performed as described in Materials and Methods. The control green fluorescent protein (GFP), which lacks subcellular targeting signals, is diffuse throughout the whole cell (Figure 1B).However, a GFP N-terminal fusion to full-length ZNF24 (aa 1–368) is exclusively localized to the nucleus (Figure 1B). This result indicates that ZNF24 is localized in the nucleus. The confirmation of ZNF24’s nuclear localization increases the likelihood that ZNF24 functions as a transcription factor.

No potential NLS was found in the amino acid sequence of ZNF24. To identify the sequence that is responsible for ZNF24 nuclear localization, we generated GFP fusion proteins of ZNF24, deleted in various portions of ZNF24, and expressed these fusion proteins in HEK293 cells. The maps of these fusion constructs are shown in Figure 1A and the corresponding fluorescence images of these constructs are shown in respectively in Figure 1B. The 161-368-GFP (aa 161-368) fusion proteins remained exclusively in the nucleus (Figure 1B). However, GFP fusion to N-terminal of ZNF24 (aa 1-51, 1-200, 1-250, 161-250), which lacks the ZFs from C-terminus, was diffuse throughout the whole cell, suggesting that the NLS sequence is within the ZF region and the ZFs are necessary for ZNF24 nuclear localization (Figure 1B). On the other hand, the molecular weight of the mutants of 1-51-GFP and 161-250-GFP are about 35kDa and 39kDa, respectively. Small proteins (less than 40 kDa) can usually pass through the nuclear pore by the free diffusion mechanism [17]. Hence, it is plausible for these mutants to enter the nucleus by simple diffusion. These visual differences were confirmed by the quantification of the nuclear/cytoplasmic fluorescence intensity (Fn/c) ratios shown in Figure 1C.

### The first and second ZFs are necessary and sufficient for ZNF24 nuclear localization

**Figure 2:**
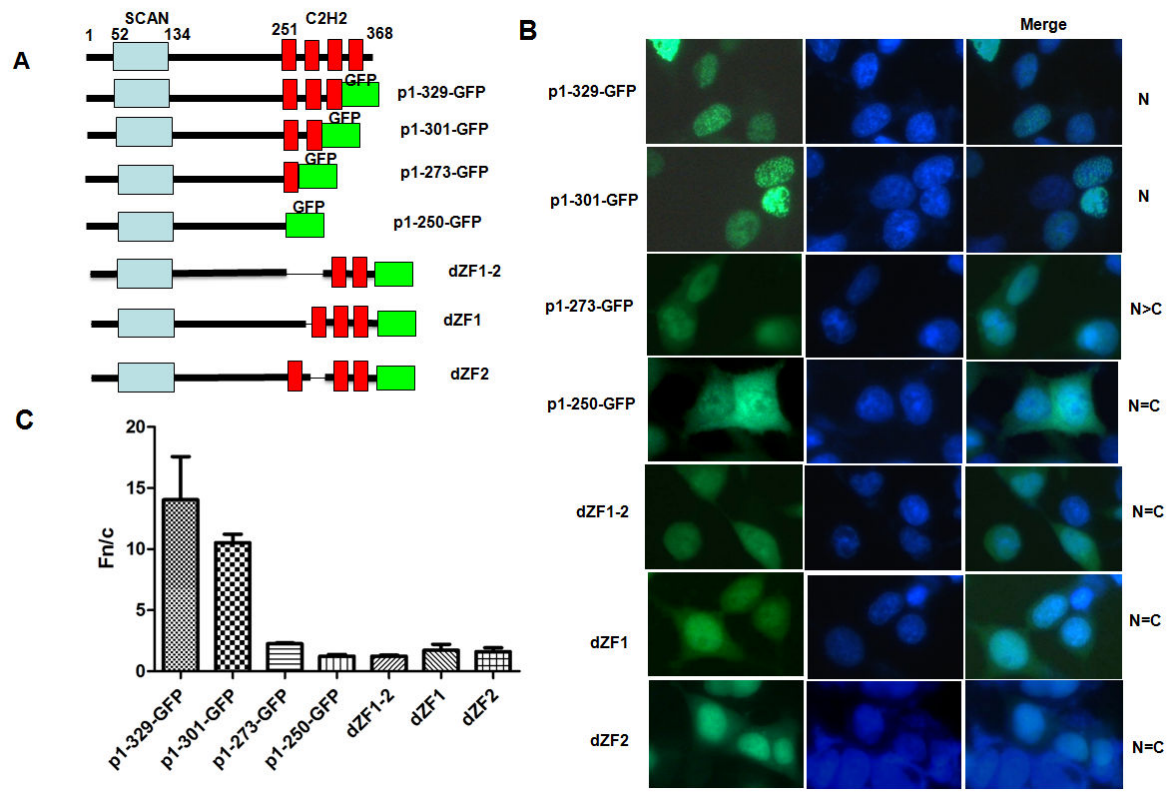
The first and second zinc fingers are necessary for efficient nuclear localization. (A) Schematic of different ZNF24-GFP deletions constructs. The shaded boxes represent features described in Figure 1. (B) Subcellular localization of the different constructs. The indicated constructs were transfected into HEK293 cells was conducted as described in Figure 1. DAPI stain is shown to delineate nuclear boundaries (blue). Patterns of localization are summarized on the right by observing 100 transfected cells from 10 to 15 independent fields. N, exclusively nuclear; N=C, most cells show both nuclear and cytoplasmic localization with similar staining intensity in the nucleus and cytoplasm; N>C, most cells show both nuclear and cytoplasmic localization with brighter staining intensity in the nucleus. (C) Results (mean±S.E.M., n=100) for quantitative analysis of images to determine the nuclear to cytoplasmic fluorescence ratio (Fn/c).

 We next carried out experiments to further delimit the NLS within the ZF region. This region consists of four Krüppel zinc finger motifs that are highly homologous to each other. We evaluated the contribution of each zinc finger motif to nuclear localization. A series of mutants were generated that deleted one to four zinc fingers. Figure 2A illustrates a schematic of these deletions. Deletion of the ZF4 (p1-329-GFP) or the ZF3-4 (p1-301-GFP) had no effect on the nuclear localization of ZNF24 (Figure 2B). However, deletion of the ZF2-4(p1-273-GFP) led to both nuclear and cytoplasmic localization with brighter staining intensity in the nucleus. Deletion of whole ZF region (p1-250-GFP) led to diffusively present throughout the cell with similar staining intensity in the nucleus and cytoplasm (Figure 2B). These data suggest that ZF1-2 (aa 251 to 301) is necessary for efficient nuclear localization of ZNF24. 

To further verify this notion, we made an internal deletion of the ZF1-2 region (Figure 2A, dZF1-2) and found that indeed all of the cells showed both nuclear and cytoplasmic localization of the mutant protein (Figure 2B, dZF1-2). To further distinguish the requirement of ZF1 from ZF2 for ZNF24 nuclear localization, we individually deleted these two ZFs (Figure 2A, dZF1 and dZF2). Surprisingly, when either of the ZFs was deleted, only few cells showed both nuclear and cytoplasmic localization of the mutant protein with brighter staining intensity in the nucleus (Figure 2B, dZF1 and dZF2) and most cells showed both nuclear and cytoplasmic localization with similar staining intensity. As in Figure 1C, the Fn/c ratios determined for each of these mutants confirmed the visual phenotypes observed (Figure 2C). This finding suggests that consecutive ZF1-2 is critical for nuclear localization of ZNF24. 

**Figure 3:**
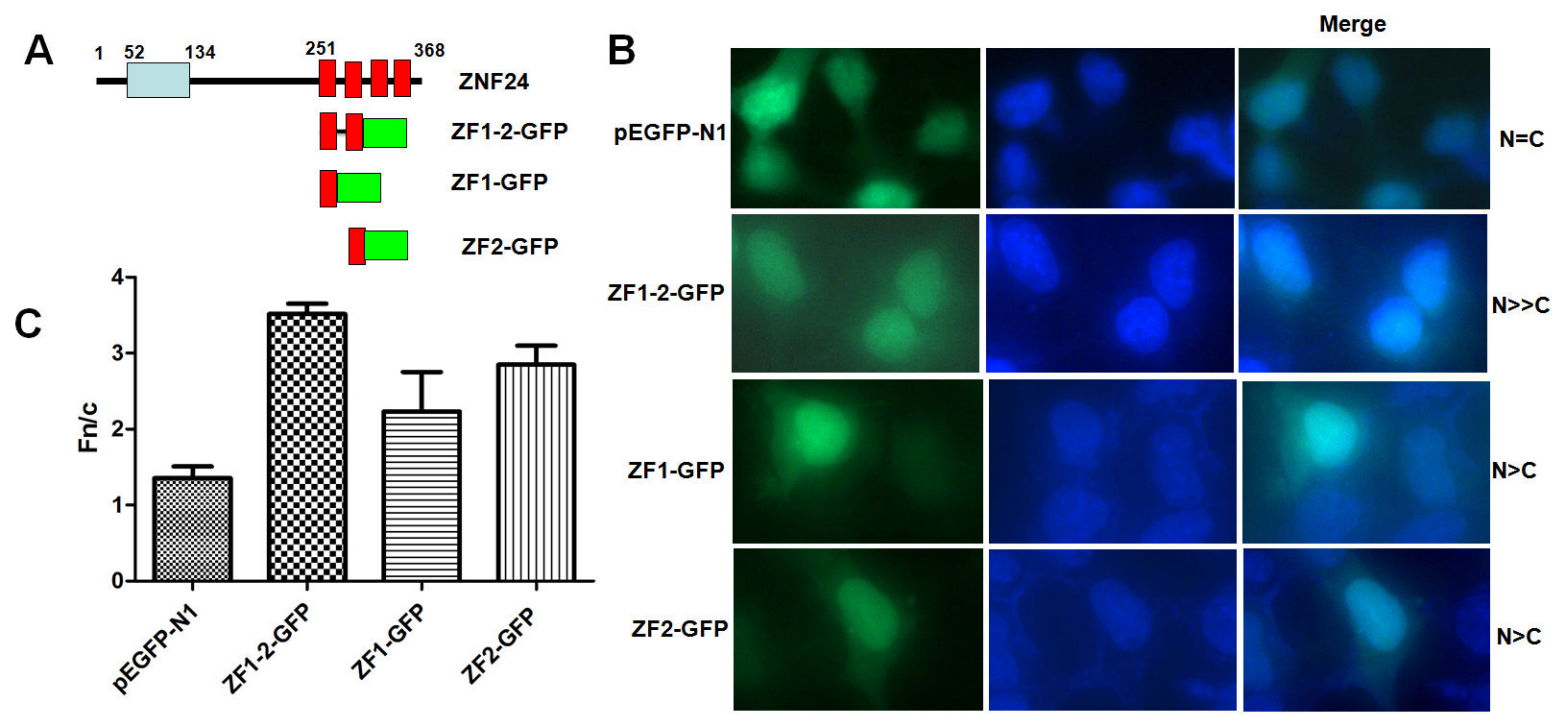
The first and second zinc fingers are sufficient for nuclear localization. (A) The ZF1-2- GFP construct is a fusion of the first and second zinc fingers to GFP. ZF1-GFP is a fusion of the first zinc finger to GFP. ZF2-GFP is a fusion of the second zinc finger to GFP. HEK293 cells were transfected with the indicated constructs, and direct immunofluorescence was conducted as described in Figure 1. (B) The subcellular localization of each mutant is summarized on the right. Representative cells from each construct are shown. (C) Results (mean±S.E.M., n=100) for quantitative analysis of images to determine the nuclear to cytoplasmic fluorescence ratio (Fn/c).

We next determined if the ZF1-2 was sufficient for nuclear localization. The ZF1-2 (aa 251-301) was fused to GFP (ZF1-2-GFP see Figure 3A) and examined for subcellular distribution. In contrast to GFP, which was distributed almost equally between the nucleus and cytoplasm, ZF1-2-GFP fusion proteins were predominantly localized to the nucleus in both HEK293 (Figure 3B) and HeLa cells (data not shown). Surprisingly, ZF1-GFP or ZF2-GFP fusion proteins were localized to the nucleus and cytoplasm with brighter staining intensity in the nucleus (Figure 3B). Figure 3C quantifies the Fn/c ratios of ZF1-2-GFP、ZF1-GFP and ZF2-GFP and confirms the visual phenotypes seen in Figure 3B. Taken together, these results demonstrate that the ZF1-2 of ZNF24 encode an NLS that is both necessary and sufficient for nuclear localization.

**Figure 4:**
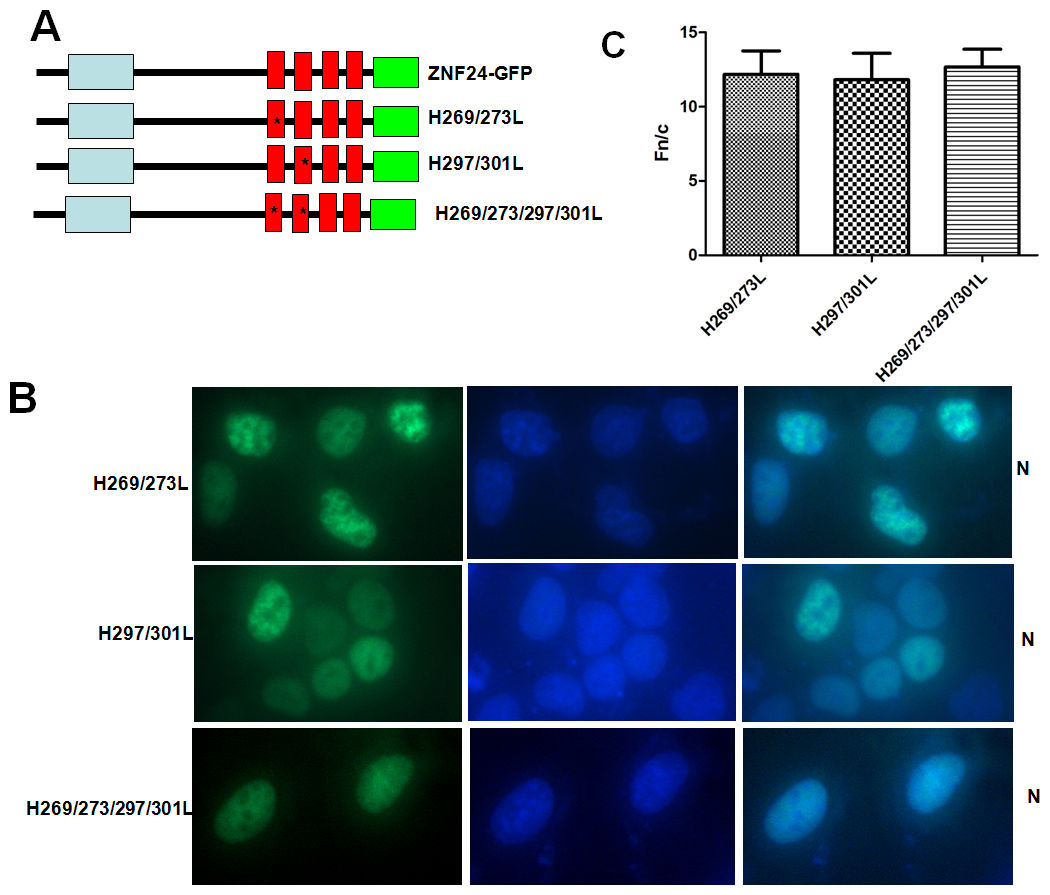
The binding to zinc ions by ZF1 or/and ZF2 is not required for ZNF24 nuclear localization. (A) Schematic of different ZNF24-GFP mutant constructs and are denoted by an asterisk. The zinc-chelating histidines of ZF1 or/and ZF2 are mutated to leucine, respectively. The shaded boxes represent the same features described in Figure 1. (B) Subcellular localization of mutant ZNF24-GFPs. Indirect immunofluoresence was conducted on the indicated constructs and analyzed as described in Figure 1. Patterns of localization are summarized on the left. (C) Results (mean±S.E.M., n=100) for quantitative analysis of images to determine the nuclear to cytoplasmic fluorescence ratio (Fn/c).

The major biochemical function of the ZFs in the nucleus is to contact the target gene promoter DNA in a Zn^2+^-binding-dependent manner[30,31]. The ZF motif of ZNF24 contains two conserved Cys and two conserved His residues. To determine whether these bindings are required for the nuclear localization of ZNF24, we individually disrupted the Zn^2+^-binding motifs (H269/273L, H297/301L and H269/273/297/301L) in ZF1 or/and ZF2 in the full-length context according for the reference[28], and examined the localization of the mutants in the cells (Figure 4). Surprisingly, none of these mutations affected the nuclear localization of ZNF24. Figure 4C quantifies the Fn/c ratios of these mutants (H269/273L、H297/301L and H269/273/297/301L) and confirms the visual phenotypes seen in Figure 4B. These results suggest that the ZFs regulate ZNF24 nuclear localization through a mechanism other than the Zn^2+^-dependent DNA binding or the tertiary zinc finger structure.

### The lysine 286 and arginine 290 residues within the first and second ZF region are critical for the nuclear localization of ZNF24

**Figure 5:**
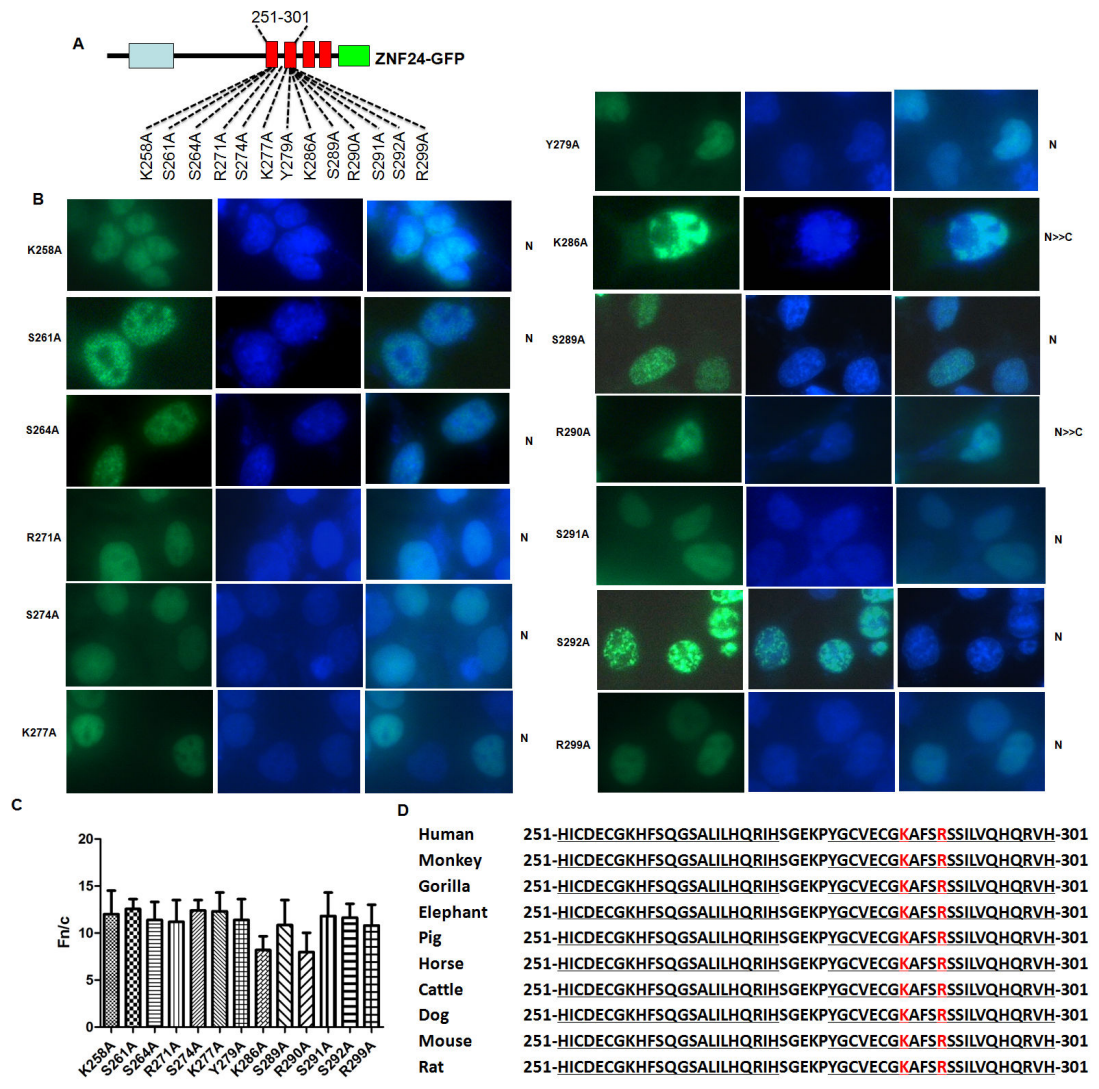
The lysine 286(K286) and arginine 290(R290) residues within the first and second ZF region are critical for the nuclear localization of ZNF24. (A) Diagram of the ZNF24 mutants with point mutation of the lysine(K), argine(R), serines (S), threonines (T) or lysines (K) to alanines (A) within the first and second ZF region as indicated. (B) The protein localization was analyzed similarly as in Figure 1. Patterns of localization are summarized on the left. (C) Results (mean±S.E.M., n=100) for quantitative analysis of images to determine the nuclear to cytoplasmic fluorescence ratio (Fn/c). (D) Conservation of the first and second ZF region of ZNF24 among the species.

The lysine and arginine residues are important for NLS activity[32]. The primary amino acid sequence of ZNF24 zinc fingers contains a total of 6 basic residues in the ZF1 and ZF2 region; two and four basic residues are present in the first and second zinc fingers, respectively (Figure 5A). We therefore sequentially replaced each lysine and arginine in ZF1 and ZF2 with alanine to investigate which amino acid residues are essential for the ZNF24 NLS. Figure 5A indicates the zinc finger amino acid sequences of these mutant proteins. The mutation of basic residues 286 or 290 to alanine (K286A or R290A) resulted in a partial mislocalization of the protein to the cytoplasm, whereas the nuclear localization of the other mutants did not significantly change (Figure 5B). Figure 5C quantifies the Fn/c ratios of these mutants and confirms the visual phenotypes seen in Figure 5B. Importantly, these residues are completely conserved across the species (Figure 5D). These results indicate that the basic residues 286 and 290 within the ZF1 and ZF2 region play an important role in nuclear localization.

 To further identify critical residues within the ZF1 and ZF2 region, we further mutated the serine, threonine or tyrosine residues within this region (Figure 5A), since these residues were potential targets for posttranslational modifications or interaction with importins that would contribute to nuclear transport[25,33,34]. However, we tested cellular localization of the mutants and found that none of the mutations affected the nuclear localization of ZNF24 (Figure 5B). 

### The C-terminal ZF regions are critical for ZNF24 interaction with importin-β

**Figure 6:**
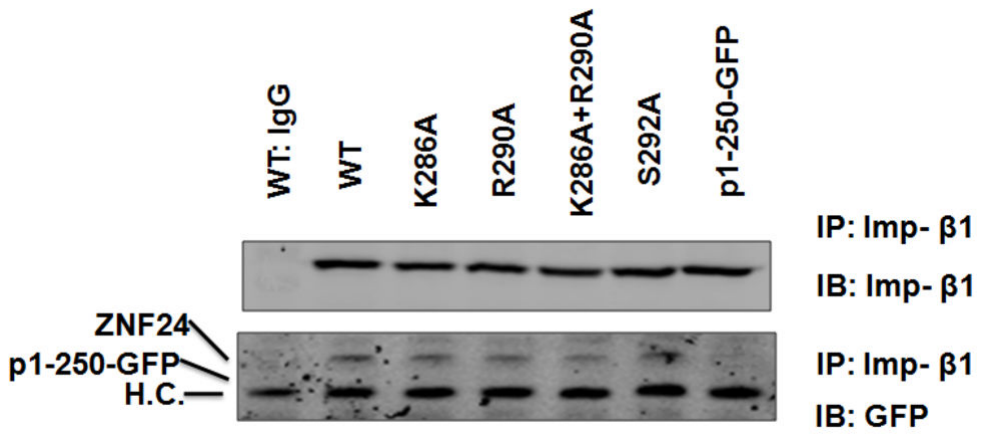
The ZF regions are essential for ZNF24 binding to importin-β1. GFP-tagged ZNF24 and mutants were transiently expressed and co-IPed with importin-β1 antibody and blotted with anti-importin-β1 or anti-GFP. Positions of the ZNF24 proteins are marked on the left side of the blots (H.C., IgG heavy chain).

Transcription factors interact with the importin proteins (importin-β1 in most cases and importin-α/β1 complex in other cases) through their NLS(s) and are then shuttled into the nucleus[28]. To test if the K286, R290 and/or the ZF region are important for ZNF24 interaction with importin-β, indicated GFP-tagged ZNF24 proteins were transiently expressed in HEK293 cells and co-IPed with importin-β (Figure 6). Clearly, ZNF24 (WT) interacted with importin-β, whereas the negative control IgG did not. This interaction was completely abolished when the ZF region was deleted (p1-250-GFP), whereas mutation of K286 or/and R290 did not affect the association. These results suggest that the C-terminal ZF region is critical for ZNF24 interaction with importin-β during its nuclear translocation, and the K286 and R290 residues may use a different mechanism to regulate ZNF24 nuclear localization. 

### The first and second ZFs are essential for transactivity of the β-Catenin

**Figure 7:**
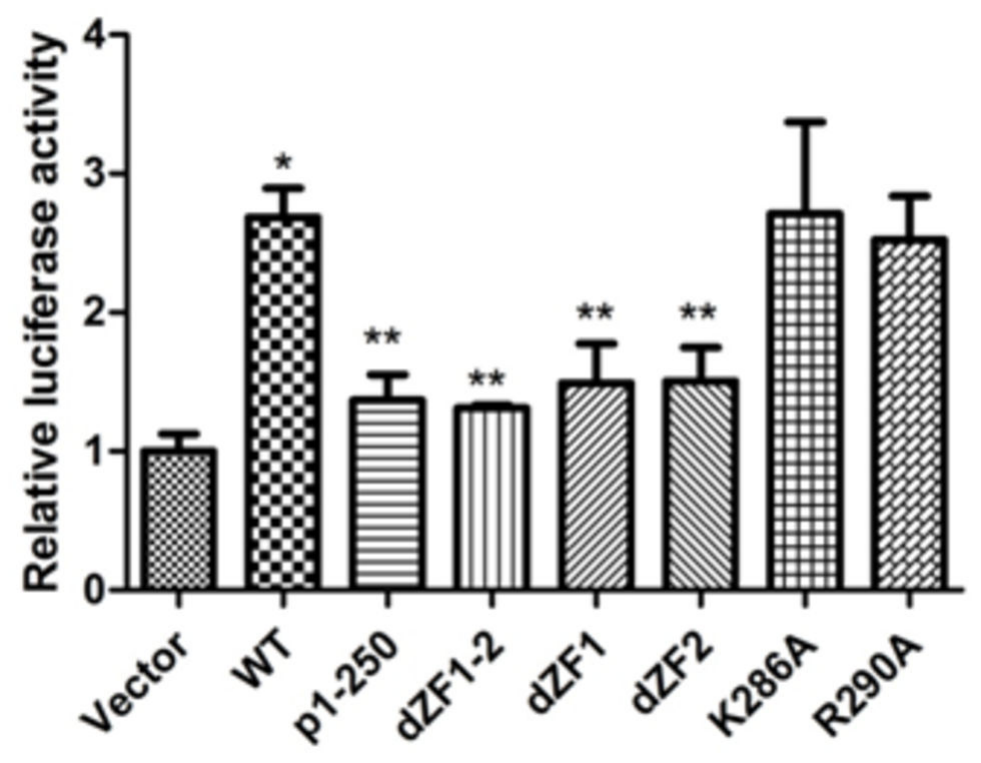
The first and second zinc fingers are essential for its regulation of transcriptional targets. HeLa cells were co-transfected with ZNF24 or its mutant constructs along with β-Catenin promoter reporter, and luciferase activities were measured after 48 h. Shown was the mean + S.E. of at least three independent experiments. *P < 0.05 compared to Vector and **P < 0.05 compared to WT.

We next examined the effect of ZNF24 nuclear localization on the transcriptional activity of ZNF24 toward the β-Catenin (CTNNB1) promoter. Using promoter luciferase reporter assays, we found that the wild-type ZNF24 enhanced the CTNNB1 promoter activity by ~2.7-fold (Figure 7, compare WT with Vector). In contrast, when the ZF region was missing, the ZNF24 mutants totally failed to activate the CTNNB1 promoter (Figure 7, compare dZF1-2 or p1-250 with WT). The K286A and R290A mutants showed no change in its transactivation function (Figure 7, compare K286A, R290A with WT). Taken together, these results suggest that the first and second ZFs that control the nuclear localization of ZNF24 are essential for ZNF24 regulation of its transcriptional target genes.

## Discussion

In this paper, we determined the NLS responsible for the nuclear localization of ZNF24. The first and second ZFs are necessary for efficient nuclear localization; deletion of ZF1 or ZF2 results in partial loss of nuclear targeting (Figure 2B), whereas deletion of ZF1-2 resulted in the nuclear and cytoplasmic localization (Figure 2B). dZF1-2 does not reduce nuclear accumulation as dramatically as the 1-250 truncation did (Figure 2). It suggests that the first and second ZFs are not entirely responsible for nuclear accumulation but important for nuclear localization of ZNF24.The first and second ZFs were sufficient to direct a heterologous protein to the nucleus (Figure 3). None of histidine to leucine mutations in the ZF1 or/and ZF2 affected the nuclear localization of ZNF24; the ability of the zinc fingers to target ZNF24 to the nucleus is independent of Zn^2+^-dependent DNA binding (Figure 4B). 

Our results also demonstrate that the ZF region is also critical for the nuclear localization of ZNF24 is consistent with previous reports that the ZFs of many ZF-containing transcription factors play an additional role as NLSs [18]. Among these transcription factors are the C_2_H_2_-type ZF transcription factors including KLF1[32], KLF8[28], ZIC3[35], SP1[36] and Zif268[37]. However, these proteins appear to have different sequence requirements for nuclear localization. For example, the NLS of ZIC3 was delimited to the second and third (of three) ZFs[35], whereas the first and second (of three) ZFs of KLF8 was sufficient for nuclear localization [28]. By contrast, all three ZFs are necessary for efficient nuclear localization of KLF1[32], SP1[36] and Zif268[37]. Our results suggest that the presence of ZF1-2 is required for the nuclear localization of ZNF24, since removal of ZF1-2 inhibited the nuclear localization of ZNF24 and removal of ZF3 or ZF4 did not affect the nuclear localization of ZNF24. Within the required ZFs, however, the zinc-binding motifs are unessential, suggesting that the ZF-mediated nuclear localization of ZNF24 does not require zinc-dependent DNA binding, which is consistent with the case of KLF1[32]and KLF8[28], but is in contrast with the case of Zif268 whose nuclear localization depends on the Zn^2+^-binding [37]. The ZFs have been shown to mediate the interaction of KLF1 with importin-α and importin-β, although whether this interaction requires the zinc-binding motifs or the DNA-contact motifs has not been examined[38].Nevertheless, our results suggest that the ZF1-2 of ZNF24 play a role in both the nuclear localization and DNA binding, whereas the major role for the ZF3-4 may be DNA binding or other. 

Our results also demonstrate that the basic residues 286 and 290 within the zinc fingers are a critical determinant for nuclear localization. Mutations of the residues 286 or 290 resulted in partial accumulation in the cytoplasm. It is worth investigating how these two residues cooperate to regulate the nuclear localization of ZNF24.Basic residues within the zinc fingers could function in several ways to affect nuclear localization. DNA and RNA binding domains are thought to function as nuclear retention signals based on their ability to bind DNA[18]. This is consistent with a survey showing that the NLS of ~70% of nucleic acid-binding proteins are coincident with the nucleic-acid binding domain [18]. Biophysical studies demonstrate that electrostatic interactions between positively charged basic residues and the negatively charged phosphate DNA backbone are the predominant mediators of nucleic acid binding [39]. Therefore, the basic residues of ZNF24 could mediate nonspecific interactions with DNA, leading to nuclear accumulation. ZNF24 containing histidine to leucine mutations that disrupt the structure of ZF1 or/and ZF2 retains appropriate nuclear localization, indicating that the tertiary structure of the zinc fingers is unnecessary for nuclear localization. Binding studies demonstrate that the nonspecific associations with DNA are independent of tertiary structure, at least for zinc finger domains [40]. This is also consistent with our results demonstrating that the tertiary zinc finger structure is dispensable for nuclear localization of ZNF24. Alternatively, the basic residues within the zinc fingers might be necessary for interacting with other chromatin-associated proteins and thus co-localize to chromatin. However, this possibility is not likely because protein-protein interactions mediated by zinc fingers usually require tertiary zinc finger structure, and our results demonstrate that finger tertiary structure is dispensable for nuclear localization. There is a formal possibility that the zinc finger basic residues constitute an unusual basic-type NLS. However, most basic-type NLSs consist of a short stretch (4–6 aa) of basic residues (classical) or two smaller clusters (~4 aa) separated by 10–12 residues (bipartite) [18]. The 6 basic residues of ZNF24 are dispersed over a relatively large region (51 amino acids). Therefore, the likelihood that this sequence constitutes an atypical basic-type NLS is remote, although not impossible.

Our results also demonstrate that the first and second ZFs of ZNF24 are essential for transcription activity of CTNNB1 promoter in HeLa cells. Promoter luciferase assay indicated that the wild-type ZNF24 can increase transcription activity of CTNNB1 promoter by about 2.7 fold compared with transfecting control vector (Figure 7). This is consistent with previous reports that ZNF24 can activate β-Catenin in hepatocellular carcinoma cell lines[9]. In contrast, when the ZF region was missing, the ZNF24 mutants failed to activate the CTNNB1 promoter (Figure 7, compare dZF1-2 or p1-250 with WT). The K286A and R290A mutants showed no change in its transactivation function (Figure 7, compare K286A, R290A with WT). It is possible that the partial mislocalization of the protein to the cytoplasm (Figure 5) is so few that the nuclear localization of mutants can enhance the CTNNB1 promoter activity as wild ZNF24 do. It is worth investigating how these two residues cooperate to regulate the nuclear localization and the transactivation function of ZNF24. These results suggest that the first and second ZFs are essential for ZNF24 regulation of its transcriptional target genes.

In conclusion, our study has led to the identification of a novel, functional NLS in ZNF24. We have also shown a role for nuclear localized ZNF24 in the regulation of CTNNB1 transcriptional activity. 
